# A Brief Account of the Epidemic Dysentery, as It Occurred in Frankfort and Franklin County, Ky., during the Summer and Fall of 1849

**Published:** 1850-01

**Authors:** William C. Sneed

**Affiliations:** Frankfort, Ky.


### WESTERN JOURNAL

OF

### MEDICINE AND SURGERY.

JANUARY, 1850.

---

Art. I,—*A Brief Account of the Epidemic Dysentery, as it occurred in Frankfort and Franklin county, Ky., during the summer and fall of* 1849. By William C. Sneed, M. D., of Frankfort, Ky.

The present state of medicine may be viewed, in all its departments, as the result of observation and the record of facts as they have been contributed by the members of the profession in various countries and at various times.

The incentives which have influenced men, in the busy scenes of public life, have had their weight in prompting them to the contribution of these facts. Zeal, in the cultivation of science, and the illustration of truth, has probably as much influence in prompting to enterprize and stimulating the labors of the medical profession, as that of any other; and possibly we would not exceed the limits of truth and justice, were we to say, that no other human pursuit has been cultivated from motives of purer devotion to the cause of science, nor any sustained by a diligence more laudable, nor directed to more useful ends.— Yet it would be uncandid, and, as we consider, unnecessary, to exclude, altogether, the natural springs of human action from all participation in the motives which have led to the accumulation and display of the various and valuable stores of our profession—its science and resources.

It could not be in the power of any one man, no matter what his opportunities, nor however enlarged his understanding, nor extensive the field of his observation and enquiries, to construct and perfect a scheme of medical science equal to the comprehensive operation which that science effects. To give it scope, efficiency, and, above all, stability, has required, and, for a time to come, will require the concurrent efforts of numerous cultivators, the record of their full investigations, the full interchange of opinions, and the multiplication of facts calculated to fix the principles of the science and regulate their application. Hence the great importance of *keeping* constantly *open* depositories for the record of observations, and even speculations in medicine. In this age, science is not stationary, is defined by no positive bounds, nor restricted within any known limits, and therefore it is the privilege, and the duty of each one to contribute all in his power to aid in its extension and improvement. Thus the labors of many devoted to one end may, like light shining from various points, serve to illuminate the objects of pursuit and enquiry.

Viewed in this light, the contributions of all who aim to promote and advance the important interests of the science, acquire a relative degree of value. And even those who do not hope to instruct the informed, nor aspire to the distinction of giving a new direction to professional opinion, may yet presume to record such facts as come under their own observations, and modestly present to the profession what has seemed to themselves either of useful application, or curious and interesting.

In this class of contributors the writer is content to take his station, emulous of no greater credit or higher commendation, than to merit the privilege of acting in concert with those of his professional brethren who are endeavoring to improve, adorn, and elevate a profession which contemplates and has accomplished so much for the good of mankind.

The preceding considerations suggest themselves as the proper explanation of the motives which prompt me to offer to the profession the following remarks upon the epidemic that occurred in our town and county during the past summer and fall.

As early as the first of May, cases of diarrhea began to occur and had all the appearance of a mild form of cholera. I was frequently applied to for medical advice in such cases, and invariably relieved them by the use of small doses of calomel and opium repeated in quantity according to the violence of the attacks. The great majority of them yielded to this course of treatment within from one to four days. I found, however, that the patient always did better under the use of these remedies, by remaining in bed, and abstaining from the use of cold drinks and solid food. One of the first injunctions which I placed upon my patients was to go to bed and remain there as quiet as possible, until the bowels were entirely relieved from pain or uneasiness, and until good bilious operations were obtained. There was a peculiar symptom, attending all these cases, which I have never met with in diarrhea before. It was a peculiar and indescribable uneasiness about the bowels. The patients would express themselves as feeling as though the whole of the abdominal viscera had been torn loose and were floating in the cavity. When they would turn over in bed, or get up, it would seem as though the peritoneal sack was full of water and the viscera were floating loose in it. There was rarely ever any nausea or vomiting, but on the contrary the appetite was good and the amount of thirst not very great. In all these cases the tongue was very white, flabby, and moist. Fever rarely or never attended, and the patients would express themselves as feeling well except the loose and peculiar sensation of the bowels. The strength of the system became speedily affected, and in a few hours such a degree of prostration followed that it was with difficulty that the patient could walk about.

These cases continued to occur until about the first of June, when they assumed a rather more grave form.—The weather about this time was warm and wet—showers falling almost daily, and the heat of the sun during the day very oppressive. During the month of June, diarrhea continued to prevail, but yielded to the usual mode of treatment—not, however, so readily as previously. A few very violent cases of cholera morbus occurred in the town and county during the month, brought on mainly by the improper use of fruit and vegetables. No death occurred, however, within my knowledge, and the course of treatment mentioned previously was pursued. Early in the month of July, a few *indigenous* cases of cholera appeared in the town and proved fatal in one or two instances. The weather still continued wet and warm, and in every way favorable to the production of such cases. Up to this time no well marked case of dysentery or flux had occurred, but cases of diarrhea increased in numbers and violence, and the cases had to be met very promptly to prevent them from running into cholera of the worst form.

The first well marked cases of flux which came under my notice occurred about the third of July; one of them was a delicate female who had been delivered a few days previously and had imprudently indulged in the use of vegetables. Her case was one of great violence, and for a while put all remedies at defiance. She, however, contrary to my expectations, recovered, after having gone through a protracted course of treatment. The next case was that of one of our most useful and respectable citizens. He had been superintendent of our gas and water works, and had worked a great deal in lead, which I incline to think, had, to some extent, deranged his system and made him much more susceptible to the epidemic influence than he otherwise would have been. His case was one of great violence, and proved fatal after *over* sixty days of great suffering. I think he would have recovered, but for an accidental hemorrhage of the bowels which occurred after he had apparently nearly recovered from the epidemic.

From this time [3d of July] the epidemic increased in violence, and by the middle of the month was in almost every part of the town and county.

The disease now under consideration commenced, in many cases, with much the appearance of the diarrhea which preceded it; frequent and unseasonable calls to stool, attended with straining and tenesmus. The evacuations were generally copious at first, of a fluid character, and in some cases of a peculiar fetid smell; some times containing streaks of blood, and at other times blood in small quantities, unmixed with fecal matter.—The pulse in the early stage was seldom altered; the heat of the skin not much increased, and the tongue in many cases but little changed in its appearance. There was, in some cases, great prostration of strength and depression of spirits, the appetite indifferent and the thirst very urgent. After this train of symptoms had prevailed for a few days, there would be a fixed pain in the hypogastrium, more or less acute; the pain extending to the iliac regions, and more or less urgent along the whole course of the colon, with fullness, tension, and tenderness upon pressure. The evacuations, as these symptoms increased, become more frequent and less copious, consisting of blood and mucus, or bloody serum, resembling water in which fresh beef had been washed. Tenesmus, with suppression of urine, now would become distressing symptoms, and the patient cared for nothing but cold drinks, with which it was impossible to satisfy his desires. The tongue became white and furred, with a tremulous motion when protruded from the mouth. The skin during the violent symptoms now existing, would be hot and dry, or covered with a profuse warm perspiration: the latter was invariably a favorable symptom. The pulse varied according to the violence of the foregoing symptoms. In some it would assume a febrile quickness, without any other distinct feature ; in others it would be full, with a peculiar jerk or rythm, which indicated more violence in the attack than the former alteration. In the fatal cases the symptoms would increase in violence from day to day until death closed the scene, which usually occurred in the course of from twelve to twenty days. I have known some patients to have from twenty to forty evacuations in the twenty-four hours, which, in quantity, would be almost incredible, and of a character differing from anything of the kind I ever saw.

The period of convalescence varied very greatly; some cases recovering in a very few days, while others were protracted for weeks, and some for months. When the disease passed into the chronic form, it differed but little, if any, from ordinary sporadic dysentery in the same stage. Ulceration of the large bowels, I have no doubt, often occurred, and was the main cause of the difficulty in conducting such cases to a favorable termination. I had only one opportunity of making an inspection of the lesion produced by the disease, and that afforded but little satisfaction as to the precise condition of the parts involved. The subject was a convict, who died of a relapse, after having apparently recovered from an ordinary attack. Inspection after death showed thickening and chronic ulceration of the lower bowel up to the sygmoid flexure; above that, to the cecum, there was acute inflammation of a mild grade, and in the small bowel, for a space of six or eight feet, complete hyperemia.—The mucous coat of the bowels was as red as scarlet, but neither softened nor ulcerated. His symptoms, during his last illness, were those of congestion, and I suppose that this condition of the small bowel was the cause of his death. On his return to the hospital, he had but little of the dysenteric character in his case. His skin was cold and moist; his pulse feeble and soft; countenance restless and cadaveric; extremities cold; no pain or tenderness about the bowels, and no tympanitis. Counter-irritants, diffusible and permanent stimulants, mercurials, &c., were all successively tried, but failed to afford relief, and he succumbed in a few days.

Several other cases of nearly the same character as the foregoing came under my notice, all of which proved fatal. I have no doubt but the scalpel would have revealed the same condition of the parts as above detailed.

The proximate cause of the disease I supposed to be the same—modified in some way—which produced the diarrhea and cholera which preceded and accompanied it. The state of the atmosphere differed in no appreciable manner from other wet and warm seasons. There was nothing either in the town or county, or about any of the locations where it prevailed to the greatest extent, to which its prevalence could be attributed. Those persons who were the most prudent in their diet were no more exempt from attacks than the most imprudent. It attacked persons of all ages, of all temperaments, and all conditions of life, with equal violence. Two functions seemed to be invariably involved, viz: the skin and the liver. My own views in relation to the pathology of the disease may be briefly stated *to be portal congestion, with a pecu-*

*liar tendency to inflammation of the lower or large bowel.* How or what way this derangement is produced, I know not, nor do I think it worth the trouble to advance any speculations on the subject in this paper.

A brief detail of the principal medical applications used in the treatment of this disease must close what I have to say upon the subject. Under their proper heads I have attempted to detail my experience in the use of the remedies which I resorted to.

*Bloodletting.*—This remedy was never tested by me, for the simple reason that I did not sufficiently understand the nature of the epidemic to risk a remedy of such potency. After a careful review of my cases, I am still unable to say what effect would probably have resulted from its use. I am satisfied, however, that in many cases it would have been wholly inadmissible, while in many others, it would have been of doubtful utility.

*Mercurials.*—The combination of calomel and opium having produced such marked and happy effects in the treatment of the diarrhea which preceded the flux, it was perfectly natural that we should resort to its use in the first cases of flux that occurred. There is, and ever has been (according to the books), a great variety of opinion in regard to the use of these medicines in this disease. According to the writers on tropical dysentery, calomel, in combination with opium, conjoined with diaphoretics, is the sheet anchor in the treatment of this disease. In warm climates, and where there is great hepatic derangement, there can be no question of the great efficiency of calomel, but in this and milder climates it is not so much relied on. I am not fully prepared to give an opinion on its virtues now, after having had such an extensive opportunity of testing it. In sporadic dysentery I have found it, with opium, equal to any other remedy. In some of the cases that came under my care, it acted like a charm, and when carried to ptyalism, produced a speedy and permanent cure.

In the penitentiary in this place there occurred, during the summer, about seventy cases of this disease, all of which recovered but one, and that was a relapsed and neglected case—and they were treated entirely with calomel, alone or compounded with opium. Here the ordinary treatment of sporadic dysentery succeeded to admiration—the patients recovering rapidly and permanently, though the attacks in many cases were violent and alarming.

In private practice the same course of treatment failed to meet my expectations. I do not recollect of more than eight or ten patients who were benefitted by being subjected to the constitutional effects of this remedy. In a few it acted well, but in others it seemed rather to provoke than mitigate the disease.

*Opium.*—This remedy has been so highly applauded by medical writers on dysentery, that it would be considered presumption to say aught against its use, at this time. I suppose it is the abuse of the article which has given rise to a prejudice against its use in my own and in the minds of others. I have tried it repeatedly, as recommended by Christison and others, and have invariably been disappointed in its effects. In my first use of it, during the prevalence of the epidemic just past, I am satisfied that it was the cause of incalculable injuries to my patients. It failed to give relief to the tenesmus and pain, and I believe seriously impeded the spontaneous efforts of the system to throw off the disease. When combined with ipecac and calomel, in small doses, its effects were beneficial, and particularly so when I gave it in small doses, frequently repeated, after the bowels had been well evacuated. The form which I preferred was that of Dover's powder and sulphate of morphine, with pulv. ipecac. The acidulated tincture, with acetate of lead, was used in cases unattended by fever or pain, and had often a good effect in lessening the frequency of the operations.

*Injections.*—From the great importance attached to this remedy by medical writers, I was educated to believe them of great utility in this disease. Having found them either inefficient or hurtful, I soon discontinued their use, except in some peculiar cases. With most patients, the introduction of the pipe more than counterbalanced the soothing effect of the injection. I have rarely seen the injection retained more than a few minutes, no matter how small in volume it might be—and when passed off, was attended invariably by tenesmus and straining. At the suggestion of Dr. Drake, I used in the latter part of the season injections composed of cold ice-water, with sugar of lead, and found that they afforded more relief than any other form I had used. As a substitute, I used in some cases opium suppositories with benefit, but I found more good to result from the use of warm fomentations to the parts, or bladders filled with hot water applied to the hypogastric region. The soothing effects produced by these applications were soon acknowledged by the patients, and often followed by sweet repose of considerable duration.

*Mustard plasters* to the sacrum, followed by warm poultices had a very soothing effect in some cases.

*Blisters.*—I used blisters to the abdomen in a few cases but found little or no good to result from them. They were objectionable on several accounts, besides the pain which they produced when the patient would have to use the chamber vessel, they were often very annoying in consequence of the stranguary caused by the absorption of the essential oil. Micturition was almost invariably difficult and painful in consequence of the irritation of the rectum, extending to the neck of the bladder, and when this part was subjected to any additional irritation, the pain was almost insupportable. Pustulation with ung. tart. ant. or croton oil over the hypogastric region seemed to be beneficial in some chronic cases.

*Local bloodletting*, by the use of cups and leeches, seemed to have little or no influence in checking the disease, and was but seldom used.

The plan of treatment which proved most successful was about as follows: when called to a case of well marked flux, I first gave a dose composed of calomel, ipecac and rhubarb, in quantity proportioned to the age and constitution of the patient. After this had acted well upon the bowels, emptying them of all crudities and feculent accumulations, I gave an anodyne, composed of pulv. Doveri or morphine and pulv. ipecac. This I would repeat two or three times at intervals of four or six hours, and at the expiration of the first twenty-four or thirty hours, I would give another purgative dose of the same composition as the first, and repeat in six hours unless free bilious discharges were produced. When the bowels were well emptied and the liver sufficiently acted upon, I would repeat the anodyne until tenesmus, tormina, and all other unpleasant symptoms were either suspended or mitigated. I found that the pulv. ipecac, combined with the purgative and sedative in large quantities, would be well borne, and had a powerful effect in determining to the surface, which was always a desirable object to be obtained. I may here remark that no case of mine proved fatal, where the skin could be sufficiently acted upon. I was always prepared to give a favorable prognosis when this function could be properly excited. If the discharges still persisted, and the tenesmus and tormina continued to be distressing, I gave, with decided good effects, "Hope's Mixture." In my first use of this remedy, I was disappointed, but by increasing the quantity of the opium, and adding about one-fifth or sixth of wine of ipecac. to the mixture, I found the most decided good effects to result from its use. To adult patients I gave a table-spoonful every hour, until its good effects were produced. With some it would be extremely grateful, quieting the stomach, relieving the pain and tenesmus in the large bowel, and acting upon the skin in a most happy manner. Its effect upon the liver in many cases was well marked, but in most cases its principle action was to the skin. In the latter stages of an attack, I occasionally gave astringents, both by the mouth and by enemas, but I placed but little reliance on their use. Where there was great prostration, with general debility, in the latter stages, tonics were used with good effect—the best of which were quinine, combined with pulv. Doveri or morphine, and good Port or Madeira wine. I used, in some cases, salts and laudanum combined, after the plan recommended in the books, but I found no good results from their use, which were not more speedily obtained by the plan above detailed. I might give additional interest to this paper by detailing at length some of the most interesting cases that c me under my treatment, but as my object is to be brief, I must decline further details.

Relapses were not frequent, but always dangerous, and oftener occurred from imprudence in eating than from all other causes. It was found best in all cases to keep the patient, from the beginning of his attack to its close on mild articles of food, and not permit the use of cold drinks of any kind. Ice water and every other cold drink had a decided tendency to aggravate the disease, without affording the patient the least comfort, even for the moment while partaking of them.

I have, in this paper, aimed at nothing but a faithful detail of the facts and observations which presented themselves during the epidemic; and submit them, with the request that you make any use of them which may seem to you proper, or best for the good of the profession.

Frankfort, December 1, 1849.

